# By Binding CD80 and CD86, the Vaccinia Virus M2 Protein Blocks Their Interactions with both CD28 and CTLA4 and Potentiates CD80 Binding to PD-L1

**DOI:** 10.1128/JVI.00207-19

**Published:** 2019-05-15

**Authors:** Patricia Kleinpeter, Christelle Remy-Ziller, Eline Winter, Murielle Gantzer, Virginie Nourtier, Juliette Kempf, Julie Hortelano, Doris Schmitt, Huguette Schultz, Michel Geist, Catherine Brua, Chantal Hoffmann, Yasmin Schlesinger, Dominique Villeval, Christine Thioudellet, Philippe Erbs, Johann Foloppe, Nathalie Silvestre, Laetitia Fend, Eric Quemeneur, Jean-Baptiste Marchand

**Affiliations:** a Transgene S.A., Illkirch-Graffenstaden, France; University of Illinois at Urbana Champaign

**Keywords:** B7-1, B7-2, CD80, CD86, M2 protein, immunosuppressor, oncolytic virotherapy, vaccinia virus

## Abstract

In this article we report that the M2 protein encoded by the vaccinia virus is secreted as a homo-oligomer by infected cells and binds two central costimulation molecules, CD80 (B7-1) and CD86 (B7-2). These interactions block the ligation of the two B7 proteins to both soluble CD28 and soluble cytotoxic T-lymphocyte associated protein 4 (CTLA4) but favor the binding of soluble PD-L1 to soluble CD80. M2L gene orthologues are found in several other poxviruses, and the B7-CD28/CTLA4 blocking activity has been identified for several culture supernatants of orthopoxvirus-infected cells and for a recombinant myxoma virus M2 protein homolog (i.e., Gp120-like protein, or Gp120LP). Overall, these data indicate that the M2 poxvirus family of proteins may be involved in immunosuppressive activities broader than the NF-κB inhibition already reported (R. Gedey, X. L. Jin, O. Hinthong, and J. L. Shisler, J Virol 80:8676–8685, 2006, https://doi.org/10.1128/JVI.00935-06). A Copenhagen vaccinia virus with a deletion of the nonessential M2L locus was generated and compared with its parental virus. This M2L-deleted vaccinia virus, unlike the parental virus, does not generate interference with the B7-CD28/CTLA4/PD-L1 interactions. Moreover, this deletion did not affect any key features of the virus (*in vitro* replication, oncolytic activities *in vitro* and *in vivo,* and intratumoral expression of a transgene in an immunocompetent murine model). Altogether, these first results suggest that the M2 protein has the potential to be used as a new immunosuppressive biotherapeutic and that the M2L-deleted vaccinia virus represents an attractive new oncolytic platform with an improved immunological profile.

**IMPORTANCE** The vaccinia virus harbors in its genome several genes dedicated to the inhibition of the host immune response. Among them, M2L was reported to inhibit the intracellular NF-κB pathway. We report here several new putative immunosuppressive activities of M2 protein. M2 protein is secreted and binds cornerstone costimulatory molecules (CD80/CD86). M2 binding to CD80/CD86 blocks their interaction with soluble CD28/CTLA4 but also favors the soluble PD-L1-CD80 association. These findings open the way for new investigations deciphering the immune system effects of soluble M2 protein. Moreover, a vaccinia virus with a deletion of its M2L has been generated and characterized as a new oncolytic platform. The replication and oncolytic activities of the M2L-deleted vaccinia virus are indistinguishable from those of the parental virus. More investigations are needed to characterize in detail the immune response triggered against both the tumor and the virus by this M2-defective vaccinia virus.

## Abstract (precis)

The vaccinia virus harbors in its genome several genes dedicated to the inhibition of the host immune response. Among them, M2L was reported to inhibit the intracellular NF-κB pathway. We report here several new putative immunosuppressive activities of M2 protein. M2 protein is secreted and binds cornerstone costimulatory molecules (CD80/CD86). M2 binding to CD80/CD86 blocks their interaction with soluble CD28/CTLA4 but also favors the soluble PD-L1-CD80 association. These findings open the way for new investigations deciphering the immune system effects of soluble M2 protein. Moreover, a vaccinia virus with a deletion of its M2L has been generated and characterized as a new oncolytic platform. The replication and oncolytic activities of the M2L-deleted vaccinia virus are indistinguishable from those of the parental virus. More investigations are needed to characterize in detail the immune response triggered against both the tumor and the virus by this M2-defective vaccinia virus.

## INTRODUCTION

Oncolytic viruses from several different virus families have raised great interest as one of the recent innovative immunotherapies to fight cancer (1, 2). The leading drug in this class of product is talimogene laherparepvec (Imlygic), an attenuated herpes simplex virus (HSV) encoding the human granulocyte-macrophage colony-stimulating factor (GM-CSF), used to treat unresectable melanoma with metastatic lesions (3). Other oncolytic viruses from several different families, which may express different payloads, are under clinical evaluation, with the most advanced being a GM-CSF-armed Wyeth vaccinia virus (TG6006/Pexa-Vec) currently in phase III trial for treatment of hepatocellular carcinoma (4). In addition to the direct lysis of tumoral cells, it has been demonstrated that oncolytic viruses are able to trigger both an intratumoral infiltration of immune cells and an antitumoral immune response that contribute to the global tumor growth inhibition of these products (5, 6). The immune response against the tumor can be further enhanced by combination with an immune checkpoint inhibitor (1, 7) or by expression of virus-encoded immunomodulators (8, 9) such as cytokines (e.g., GM-CSF), antibodies (9, 10), or both. Among the currently evaluated oncolytic viruses, vaccinia viruses from different strains have been developed by several academic groups and biotech companies for different oncological applications (for a review, see reference 11). These viruses carry different gene deletions to restrict their replication to tumoral cells, and they express a variety of payloads. Vaccinia virus offers several advantages such as a well-established safety profile, a large insertion capacity for vectorization of large and/or multiple transgenes, a good relative resistance to immune neutralization, and a well-established manufacturability (11, 12). About 40 out of the 250 genes of the vaccinia virus genome are well known to encode immunosuppressors that contribute altogether to hamper the antiviral immune response (13). These immunosuppressors target a wide variety of pathways by interacting with both intracellular (several NF-κB activation inhibitors) and extracellular (e.g., type I/II interferon decoy receptors) central immune mediators (13, 14). Several of these immunosuppressor genes have been eliminated during the generation of the highly immunogenic, nononcolytic, modified vaccinia virus Ankara (MVA) that is widely used as a prophylactic or therapeutic vaccine platform to fight infectious diseases and cancers. Considering that these immunosuppressive functions could negatively impact the therapeutic efficacy of oncolytic vaccinia virus, some investigators have deleted some of these genes and have demonstrated an overall improved antitumoral activity of the resulting virus (15).

We present here some evidence of new immunosuppressive functions for the M2 protein. It was shown to bind both CD80 and CD86 and to inhibit their binding to their soluble cognate receptors, i.e., CD28 and cytotoxic T-lymphocyte associated protein 4 (CTLA4). Moreover, the CD80-M2 soluble complex was demonstrated to have an increased affinity for PD-L1. A vaccinia virus with a triple deletion (TD) of thymidine kinase, ribonucleotide reductase, and M2L (TK^−^ RR^−^ M2L^−^ vaccinia virus, or VV-TD) was generated and characterized for its *in vitro* and *in vivo* replication and oncolytic activities.

## RESULTS

### Culture supernatants from vaccinia virus-infected cells inhibit the interaction of soluble CTLA4 with CD80 or CD86.

**FIG 1:**
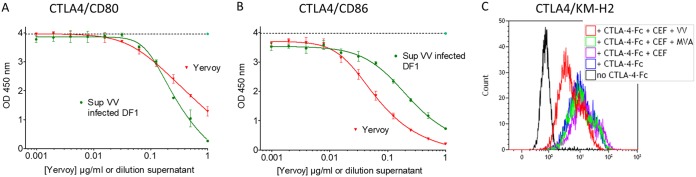
Culture supernatants of vaccinia virus-infected cells inhibit the binding of hCTLA4-Fc to CD80 or CD86. DF1 cells were infected by vaccinia virus (Copenhagen TK^−^ RR^−^) at an MOI of 0.05. Forty-eight hours after infection, culture supernatants were recovered, centrifuged, filtered, and tested by ELISA as follows. A total of 250 ng of hCTLA4-Fc was coated on an ELISA plate, and 2-fold serial dilutions of supernatant (Sup) or ipilimumab (Yervoy) (starting at 1 μg/ml) were added concomitantly with either 25 ng/ml of His-tagged hCD80-Fc (A) or 100 ng/ml hCD86-Fc (B) protein. The binding of B7 proteins to CTLA4 was monitored using an HRP-conjugated anti-His antibody. Each represented value is the mean (± standard deviation) of triplicate wells. The dotted lines correspond to the signals obtained with the undiluted supernatant of the uninfected cells. (C) CEFs were infected by vaccinia virus (Copenhagen TK^−^ RR^−^) at an MOI of 0.05. Forty-eight hours after infection, culture supernatants were recovered, centrifuged, filtered, and tested as follows. Culture supernatants were tested for their blocking activity on the hCTLA4–Fc interaction with KM-H2 cells by flow cytometry. A total of 1 × 10^5^ cells was first stained using Live/Dead Fixable Violet Dead Cell Stain kit and then incubated with PBS (no CTLA4–Fc) or with 100 ng/ml CTLA4–Fc in the presence or absence of culture supernatants from virus-infected or uninfected CEFs. CTLA4–Fc binding was detected with 5 μg/ml PE-conjugated anti-human IgG Fc and analyzed within the live singlet population. OD, optical density.

**FIG 2:**
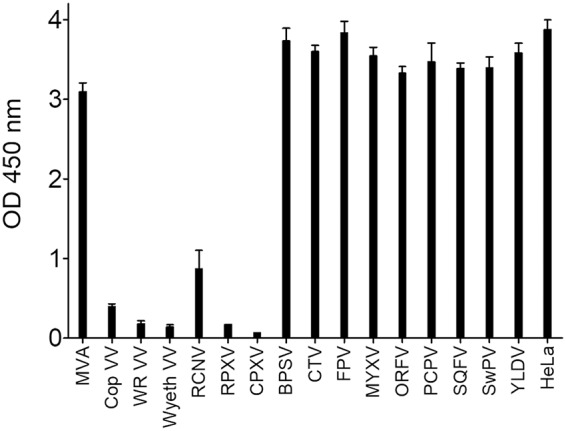
Culture supernatants of orthopoxvirus-infected HeLa cells inhibit the binding of hCTLA4 to hCD80. HeLa cells were infected at an MOI of 1 by different poxviruses, and the culture supernatants were recovered 48 h after infection. The supernatants were centrifuged, filtered on a 0.2-μm-pore-size filter to remove most of the virus, and tested for their capacity to inhibit the CD80-CTLA4 interaction as described in the legend of Fig. 1. Each represented value is the mean (± standard deviation) of triplicate wells. MVA, modified vaccinia virus Ankara; Cop VV, Copenhagen vaccinia virus; WR VV, vaccinia virus Western reserve; Wyeth VV, vaccinia virus Wyeth; RCNV, raccoonpox virus; RPXV, rabbitpox virus; CPXV, cowpox virus; BPSV, bovine papular stomatitis virus; CTV, Cotia virus; FPV, fowlpox virus; MYXV, myxoma virus; ORFV, ORF virus; PCPV, pseudocowpox virus; SQFV, squirrel fibroma virus; SWPV, Swinepox virus; YLDV, Yaba-like disease virus.

During the process of vectorization of anti-CTLA4 monoclonal antibodies (MAbs) into an oncolytic vaccinia virus, for the purpose of local delivery into the tumor of this efficient but rather toxic molecule, two enzyme-linked immunosorbent assays (ELISAs) were set up to monitor quantitatively the human CTLA4 (hCTLA4)-hCD80/hCD86 blocking activities of the vectorized MAbs. In these assays, hCTLA4 was immobilized on ELISA plates, and soluble tagged hCD80 or hCD86 was added. In this setting, any competitive molecule that will bind to either the immobilized or the soluble partner would induce a decrease of signal (competition assay). The anti-hCTLA4 MAb ipilimumab (Yervoy) and supernatant of cells infected with a nonrecombinant (empty) vaccinia virus were used as positive and negative controls, respectively. Surprisingly, the culture supernatant from DF1 cells infected with empty vaccinia virus was found to compete in a dose-dependent manner in both hCTLA4-hCD86 and hCTLA4-hCD80 assays (Fig. 1A and B), whereas the supernatant from uninfected cells had no blocking activity. Interestingly, supernatants from MVA-infected cells had no inhibiting activity on the hCTLA4-hCD80/hCD86 interactions (data not shown), indicating that this interference ability is not conserved in this virus strain which has lost several genes during its attenuation process. The interference of vaccinia virus-infected cell culture supernatant was confirmed by flow cytometry using the CD80- and CD86-positive human cell line KM-H2 (Hodgkin lymphoma [16]) and soluble recombinant CTLA4-Fc. The supernatant from vaccinia virus-infected cells inhibited the binding of CTLA4 to KM-H2 cells whereas the supernatant from MVA-infected cells had a minimal effect compared to that of the supernatant from uninfected cells (Fig. 1C). To evaluate the extent of this phenomenon, HeLa instead of DF1 cells were infected with different poxviruses (Fig. 2 and legend) at a high multiplicity of infection (MOI) (i.e., an MOI of 1) to guarantee an optimal infection and expression of viral proteins by infected cells, and the resulting culture supernatants were tested in a CD80-CTLA4 ELISA. An MOI of 1 was used here, instead of an MOI 0.01 as in the previous experiment, because some of the poxviruses do not, or only poorly, replicate in HeLa cells. Therefore, a high MOI partially compensates for this poor replication and maximizes the expression of viral proteins by infected cells. In this screening experiment, endpoint measures were performed with undiluted supernatants from infected cells. Because the different poxviruses replicate differently in HeLa cells (17), only samples demonstrating interference were considered and interpreted. Culture supernatants from cells infected with either of three strains of vaccinia virus or with three other orthopoxviruses tested (i.e., raccoonpox, rabbitpox, and cowpox) were able to interfere with the binding of hCTLA4 to hCD80 (Fig. 2). These results indicate that one or several factors secreted during infection with orthopoxviruses, except MVA, are interfering with the CTLA4-B7 pathway. This new unknown factor was called the interference factor (IF).

### IF binds to CD80 and CD86.

**FIG 3:**
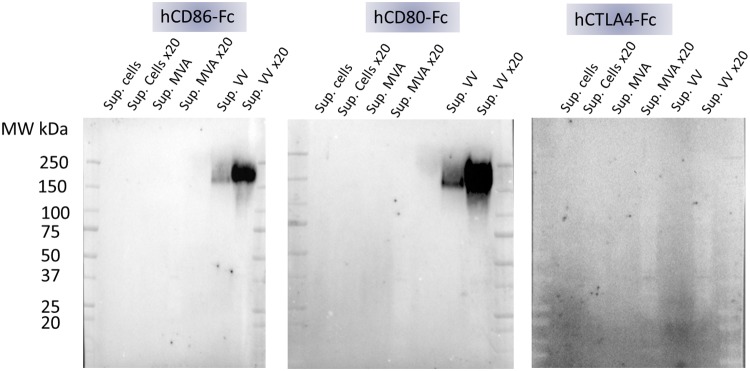
Culture supernatants of vaccinia virus-infected cells contain a complex that interacts with CD80 and CD86 but not with CTLA4. CEFs were mock infected or infected with either MVA or vaccinia virus at an MOI of 0.05. Forty-eight hours after infection, the culture supernatants were recovered and concentrated 20-fold using a 30,000-MWCO concentrator. The concentrated and unconcentrated supernatants were loaded on 4% to 15% SDS-PAGE gels, and proteins were transferred on PVDF membranes. Membranes were hybridized with 2.5 μg/ml of hCD80-Fc, hCD86-Fc, or hCTLA4-Fc. The binding of the Fc fusion proteins was monitored using an HRP-conjugated anti-Fc antibody and an enhanced chemiluminescence kit. MW, molecular weight.

We focused our efforts on the characterization of the vaccinia virus interference factor (IF). A Western blot of culture supernatants from CEFs uninfected or infected with either MVA or vaccinia virus was probed with the three components of the ELISA (namely hCD80, hCD86, and hCTLA4). CEFs were chosen since they are permissive to both vaccinia virus and MVA that do and do not produce the IF, respectively. Each protein used to probe the Western blot was a fusion with an Fc part that allows protein dimerization and detection with the same anti-Fc-conjugated antibody. The blots presented in Fig. 3 demonstrated unambiguously that a large molecule of about 200 kDa present only in the vaccinia virus-infected culture supernatants interacted with both hCD80-Fc and hCD86-Fc but not with hCTLA4-Fc. Sample reduction prior to electrophoresis completely abolished the detection of this band, indicating that intra- and/or interdisulfide bonds are necessary to maintain the IF structure involved in the CD80 and CD86 interaction (data not shown).

### IF inhibits the binding of CD80 and CD86 to soluble CTLA4 and CD28 but potentializes the binding of soluble CD80 to PD-L1.

**FIG 4:**
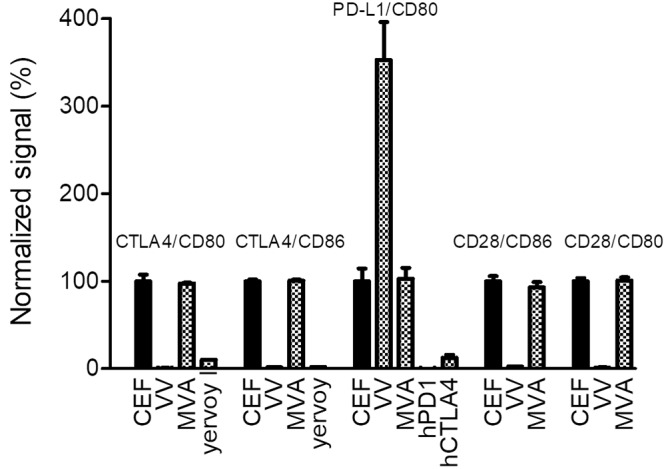
Culture supernatants of vaccinia virus-infected CEFs inhibit the binding of CTLA4 and CD28 to CD80 and CD86 but potentiate the binding of PD-L1 to CD80. Either hCTLA4-Fc, hCD28-Fc, or hPD-L1–Fc was coated on ELISA plates. Supernatants of noninfected CEFs or CEFs infected with either MVA or vaccinia virus (MOI of 0.05; 48 h postinfection) were added concomitantly with either biotinylated hCD80-Fc or hCD86-Fc (from 50 to 500 ng/ml, depending on the interaction). The binding of B7 proteins was monitored using HRP-conjugated streptavidin. Ipilimumab (Yervoy), hPD-1–Fc, and CTLA4-Fc at 10 μg/ml were used as competitors (positive controls) in some of the interactions (see graph for details). The optical density values recorded were normalized using the mean of the triplicate values of uninfected CEFs as 100% of the signal. Each represented value is the mean (± standard deviation) of three normalized measurements.

CD80 and CD86 interact with both CTLA4 and CD28, and CD80 also interacts with PD-L1. To decipher the effect of IF on each of these specific interactions, different ELISAs were set up, and undiluted culture supernatants from vaccinia virus-infected CEFs were tested. As described above, IF inhibits the interaction of hCD80 and hCD86 with hCTLA4. As shown in Fig. 4, supernatants were also able to abolish the important interaction of hCD80 or hCD86 with soluble hCD28, as expected, since CTLA4 and CD28 have overlapping CD80/CD86 binding sites (18). This CD80/CD86-CD28 inhibition makes also a lot of sense from a virus perspective to minimize or delay its clearance by the adaptive immune response. Surprisingly, the soluble hPD-L1–hCD80 interaction was increased in the presence of vaccinia virus supernatant in contrast to results with CTLA4, which competes with PD-L1 for CD80 binding. This result indicates that the IF and CTLA4 binding sites on CD80 are not completely overlapping.

### The vaccinia virus M2 protein elutes from a CD86 affinity matrix.

Based on the apparent molecular weight of ∼200 kDa and the fact that IF was not produced after MVA infection, the 37 genes that are present in the vaccinia Copenhagen strain, and not in MVA, were examined without finding any obvious candidate. Moreover, the largest protein encoded by vaccinia virus, based on its primary structure, is the DNA-dependent RNA polymerase subunit RPO147 (J6R) with a calculated mass of 147 kDa. In other words, there was no obvious viral candidate that could be linked to IF. Therefore, an experimental approach to identify IF was attempted using affinity chromatography with immobilized CD86-Fc and a 20-fold-concentrated culture supernatant of vaccinia virus-infected CEFs. Concentrated supernatant from MVA-infected cells and immobilized CTLA4-Fc were used as two negative controls (see Materials and Methods for details). The different elutions of the affinity chromatography arms were digested by trypsin, and the resulting peptides were analyzed by liquid chromatography coupled to tandem mass spectrometry (LC-MS/MS). The obtained *m/z* data were used to probe the chicken (Gallus gallus) and vaccinia virus protein data banks. The MS results allowed the unambiguous identification of the vaccinia virus M2 protein only in the sample coming from the supernatant of vaccinia virus-infected CEFs incubated with CD86-coated beads. This result agrees with the absence of the M2L gene in MVA and with the fact that M2 protein has a predicted signal peptide, making it a putative secreted protein. However, the M2 protein has a calculated molecular mass of only 25 kDa and has been reported to migrate in SDS-PAGE performed under reducing conditions as a 35-kDa glycosylated protein (19). These features are not in agreement with the 200-kDa mass of IF observed in SDS-PAGE. Nevertheless, to our knowledge, the behavior of M2 protein in SDS-PAGE under nonreducing conditions was not documented. M2 could be part of IF either as a homo- or hetero-multimeric complex with intersubunit disulfide bonds and with an apparent mass by SDS-PAGE of ∼200 kDa.

### M2L-deleted virus does not produce IF.

**FIG 5:**
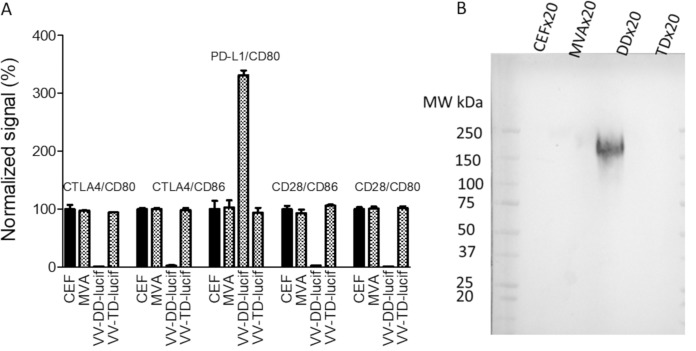
Culture supernatants of M2L-deleted vaccinia virus-infected CEFs do not interfere with the B7–CTLA4/CD28/PD-L1 interactions. (A) Culture supernatants collected from uninfected CEFs, as negative controls, or from CEFs infected with either MVA or a double-deleted (TK^−^ RR^−^, VV-DD-Luc) or a triple-deleted (TK^−^ RR^−^M2L^−^, VV-TD-Luc) Copenhagen vaccinia virus were concentrated and treated as described in the legend of Fig. 3 The blot was incubated with 2.5 μg/ml of CD80-Fc, and the binding of the fusion protein was monitored using an HRP-conjugated anti-Fc antibody and an enhanced chemiluminescence. kit. (B) The supernatants (but unconcentrated) used for the experiment described in panel A were tested in the same competition assays as described in the legend of Fig. 4. The optical density values recorded were normalized using the mean of the triplicate values of uninfected CEFs as 100% of the signal. Each represented value is the mean (± standard deviation) of three normalized measurements.

The M2L locus was used as a site of genome insertion several times in the past to generate recombinant vaccinia virus expressing different transgenes, indicating that this gene is not essential for viral replication (20). Therefore, to verify that IF contains M2, the M2L gene was disrupted in a double-deleted (DD) vaccinia virus backbone (i.e., TK^−^ RR*^−^)* encoding luciferase at the TK locus, resulting in a triple-deleted (TD) virus (i.e., M2L^−^ TK^−^ RR^−^). As demonstrated by the results shown in Fig. 5A, the culture supernatant from M2L-deleted virus-infected cells, unlike that from the parental virus, was not able to interfere with the B7-CTLA4/CD28/PD-L1 interactions. Moreover, the large complex detected by Western blotting using CD80-Fc or CD86-Fc was no longer detected in the culture supernatant of cells infected with the M2L-deleted virus (Fig. 5B). These results confirmed that M2 is at least part of the IF.

### Recombinant M2 forms a 200-kDa homo-oligomer and interferes with B7-CTLA4/CD28/PD-L1 interactions.

**FIG 6:**
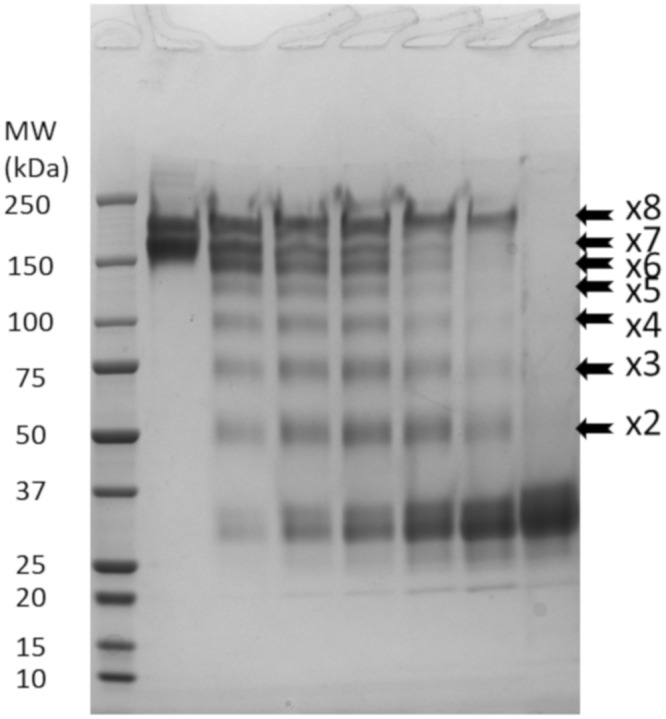
M2 protein is a homo-oligomer. SDS-PAGE of 7.5 μg of purified recombinant M2 was performed under nonreducing conditions (0 mM dithiothreitol [DTT]; first lane after the molecular weight [MW] ladder) and in the presence of increasing amounts of reducing agent (DTT) from 1 mM to 16 mM, loaded, respectively, from lanes 2 to 6. The seventh lane corresponds to the protein completely reduced by beta-mercaptoethanol. Detection was performed with Coomassie blue staining. Each arrow corresponds to the position of an intermediate product of the oligomerization. Each number indicates the putative number of protomer in the visualized band.

**FIG 7:**
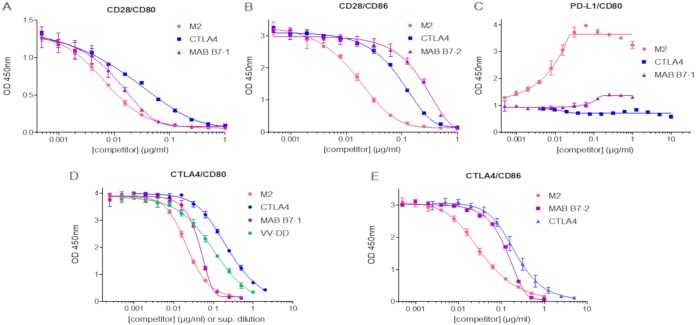
Pure recombinant M2 protein recapitulates IF activities on B7–CTLA4/CD28/PD-L1 interactions. The effect of the purified M2 protein on CD28-CD80 (A), CD28-CD86 (B), PD-L1–CD80 (C), CTLA4-CD80 (D), and CTLA4-CD86 (E) interactions was compared to those of ipilimumab (Yervoy), recombinant hCTLA4-Fc, anti-hCD80 monoclonal antibody (MAb B7-1), and anti-hCD86 monoclonal antibody (MAb B7-2) as well as with the supernatant collected from CEF cells infected with TK^−^ RR^−^ Copenhagen vaccinia virus (VV-DD) in the case of the CTLA4-CD80 interaction. Binding of His-tagged B7-Fc proteins to immobilized CTLA4-Fc, CD28-Fc, or PD-L1–Fc was monitored using an HRP-conjugated anti-His antibody. Each represented value is the mean (± standard deviation) of three measurements.

In order to investigate if other viral or cellular factors are involved in the interference of the CD80/CD86–CTLA4/CD28/PD-L1 interactions, recombinant tagged M2 was cloned and produced by transient transfection of HEK293 cells and purified by affinity chromatography to near homogeneity (no other protein was detected by MS analysis of the digested purified M2 protein). Recombinant purified M2 was analyzed by gel electrophoresis under reducing and nonreducing conditions (Fig. 6). Under reducing conditions, tagged M2 migrated slightly below the 37-kDa marker, in agreement with the published 35-kDa size for a nontagged M2 (19). Moreover, under nonreducing conditions, a band was observed at a size close to that of IF (i.e., 200-kDa area) (Fig. 6), indicating that M2 can auto-assemble into a large complex stabilized by intersubunit disulfide bonds. The use of intermediary concentrations of reducing agent allowed visualization of the transitional products of assembly between the 35-kDa and the ∼200-kDa product, suggesting the formation of a homo-hexo- to octamer. Mass spectrometry analysis under nonreducing conditions did not allow determination of the number of subunits involved in the oligomer mostly because of the large heterogeneity of glycosylation of the protomers (19) that, combined with oligomerization, makes the mass spectrometry signal very broad and impossible to interpret. The effect of purified M2 protein on the five interactions involving CD80 and CD86 was assessed by ELISA. As shown in Fig. 7, the M2 protein was able to mimic all the activities displayed by culture supernatant from vaccinia virus-infected cells. These activities were benchmarked with both the CTLA4-Fc fusion protein and CD80- or CD86-blocking MAb. In both cases, the M2 protein demonstrated superior or equal activity to these blocking molecules. Again, in the case of the CD80–PD-L1 interaction, M2 was the only component able to increase the binding of the two soluble ligands (Fig. 7). In the case of the CTLA4–CD80 interaction, supernatant from vaccinia virus-infected CEFs was incorporated in the ELISA, and the data generated with the recombinant M2 protein were used for a standard curve to back-calculate the actual concentration of M2 produced by an infection. Forty-eight hours after infection the concentration of M2 in the culture supernatant was approximately 0.25 μg/ml. These results demonstrate that, in the absence of any other cell or vaccinia virus protein, recombinant M2 recapitulates both the electrophoresis behavior and the CD80/CD86–CTLA4/CD28/PD-L1 interference activities of IF. In other words, M2 is IF.

### Recombinant M2 protein binds to murine/human CD80 and murine/human CD86.

**FIG 8:**
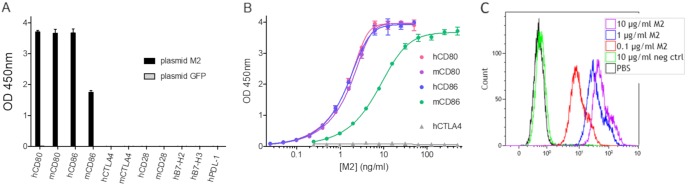
Recombinant M2 binds to human and murine soluble CD80 and CD86 proteins and to human CD80- and CD86-positive cells. (A) The following recombinant Fc fusion proteins from the human B7 family were coated on ELISA plates (0.5 μg/ml): human CD80 (hCD80), mouse CD80 (mCD80), hCD86, mCD86, hCTLA4, mCTLA4, hCD28, mCD28, hB7-H2, hB7-H3, and hPD-L1. Undiluted culture supernatants of HEK293 cells transfected with either “plasmid M2” encoding a Flag-tagged M2, or with “plasmid GFP” encoding green fluorescent protein (as a negative control) were added. Bound recombinant protein was detected using an HRP-conjugated anti-Flag antibody. Each represented value is the mean (± standard deviation) of triplicate wells. (B) Human or mouse CD80-Fc and CD86-Fc and human CTLA4-Fc were coated at 1 μg/ml on ELISA plates before the addition of serial dilutions of purified Flag-tagged M2 protein. Bound recombinant protein was detected with an anti-Flag-HRP antibody. Each represented value is the mean (± standard deviation) of triplicate wells. (C) M2 interaction with human CD80^+^ CD86^+^ cells was assessed by flow cytometry. A total of 3 × 10^5^ KM-H2 cells was first stained using a Live/Dead Fixable Violet Dead Cell Stain kit and then incubated with the indicated concentrations of recombinant FLAG-tagged M2, irrelevant FLAG-tagged protein (negative control), or PBS. M2 binding was detected using 1 μg/ml PE-conjugated anti-FLAG tag and analyzed within the live singlet population.

Recombinant tagged M2 protein was used to investigate in more detail the interactions between M2 and several other members of the human B7 family using ELISAs. As shown in Fig. 8A, M2 protein interacted with both human and murine CD80 and CD86 but with no other protein of the B7 family tested (Fig. 8A). The signal obtained with murine CD86 was lower than the one observed with human CD86. To document the binding of M2 to CD80 and CD86, dose-response curves were generated for both species. The results presented in Fig. 8B demonstrate clearly that vaccinia virus M2 has the same apparent affinities for murine CD80, human CD80, and human CD86. However, the apparent affinity for murine CD86 seemed to be lower than that for its human or its murine CD80 counterpart. The binding of M2 to cell surface human CD80 and CD86 was also verified by staining KM-H2 cells with recombinant tagged M2 (Fig. 8C).

### M2 ortholog from myxoma virus interacts with hCD86.

**FIG 9:**
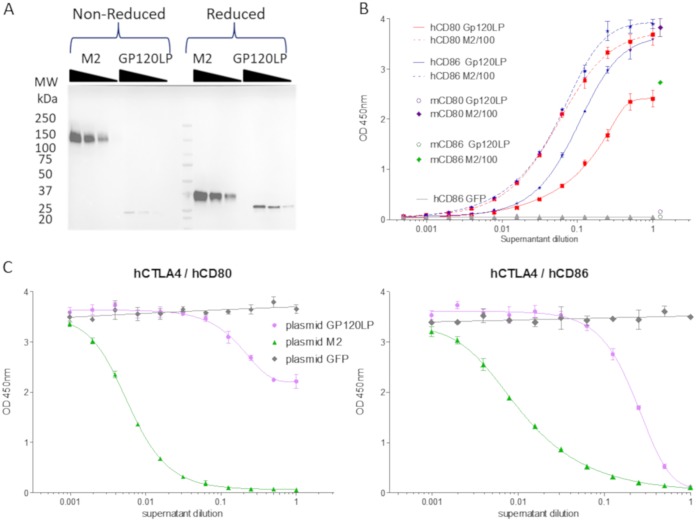
The Gp120LP myxoma virus M2 homolog protein binds to human CD80 and CD86 proteins but not to their murine counterparts. (A) FLAG-tagged recombinant Gp120LP protein, a myxoma virus homolog of vaccinia virus M2, was produced in supernatant of transiently transfected HEK293 cells. Different volumes (10, 5, and 2.5 μl) of the culture supernatants of cells transfected by a plasmid encoding either M2 or Gp120LP were loaded on 4% to 15% SDS-PAGE gels and transferred on PVDF membranes. The recombinant proteins were detected using an anti-Flag-HRP antibody and an enhanced chemiluminescence kit. MW, molecular weight. (B) The same supernatants were assessed for murine and human CD80-Fc and CD86-Fc binding as described in the legend of Fig. 8B. Undiluted supernatant of cells transfected with a plasmid encoding Gp120LP or 100-fold-diluted culture supernatant of cells transfected with a plasmid encoding M2 was added to plates coated (at 1 μg/ml) with human and murine CD80-Fc and CD86-Fc. Samples were then diluted (2-fold serial dilution) in blocking solution, and bound recombinant protein was detected by an HRP-conjugated anti-Flag antibody. Each represented value is the mean (± standard deviation) of triplicate wells. The binding levels to human B7 proteins are represented as full curves with either dashed lines (supernatant of cells transfected with a plasmid encoding M2 diluted 100-fold as starting material) or plain lines (supernatants of cells transfected with a plasmid encoding Gp120LP or GFP). For the sake of clarity, only the first points are presented for the binding to murine B7 proteins of the 100-fold-diluted supernatant of cells transfected with a plasmid encoding M2. (C) Supernatants were also tested for their ability to inhibit the hCTLA4-hCD80/hCD86 interaction in the same assay as described in the legend of Fig. 1 Supernatant of untransfected cells or cells transfected with a plasmid encoding GFP were used as negative controls. Each represented value is the mean (± standard deviation) of triplicate wells.

The M2L gene from vaccinia virus has orthologues in others chordopoxviruses (Table 1). To investigate if the CD80/CD86 binding activity is conserved along the poxvirus family, the distant M2L ortholog M154L from myxoma virus was cloned and expressed in HEK293 cells as a tagged protein. M154L was chosen because of its low identity with M2 from vaccinia virus (50% at the protein level) (Table 1) and because the culture supernatant of HeLa cells infected by myxoma virus did not show any interference activity with respect to the hCD80-hCTLA4 interaction (Fig. 2). HEK293 cells were transiently transfected with plasmids encoding either FLAG-tagged vaccinia virus M2 or Gp120-like protein (Gp120LP; product of M154L gene), and culture supernatants were recovered and assayed for levels of expression, CD80 and CD86 binding, and CTLA4-CD80 or -CD86 interaction inhibition. Gp120LP was expressed at a lower level than M2 and did not show a shift in migration when loaded on SDS-PAGE gels under nonreducing conditions, indicating either a monomeric state or an oligomeric state without intermolecular disulfide bonds (Fig. 9A). However, the culture supernatant containing Gp120LP displayed some binding activities toward hCD86 and hCD80 in ELISAs, but with a 50% effective concentration (EC_50_) more than 100-fold lower than that of the corresponding supernatants containing the tagged vaccinia virus M2 protein (Fig. 9B; note that the results presented were generated with undiluted supernatant [Gp120LP] and supernatant diluted 100-fold [M2]). Despite this drastic decrease in hCD80 and hCD86 binding, Gp120LP is able to block totally the interaction of hCD86-Fc with hCTLA4-Fc and at least partially the interaction of hCD80-Fc with hCTLA4-Fc (Fig. 9C). To fully assess the binding and blocking ability of Gp120LP, rabbit CD80 and CD86 should be used as rabbit is the natural host of this virus. All together, these results demonstrate that the CD80/CD86 binding and blocking activities of different M2 proteins are represented among different poxviruses.

**TABLE 1:** List of poxviruses with identified M2L orthologous genes

Genus or family	Poxvirus name	GenBank accession no.	% protein identity^*a*^
*Orthopoxvirus*	Vaccinia virus	AAA48004.1	100
	Rabbitpox virus	AAS49736.1	100
	Horsepox virus	ABH08137.1	99
	Cowpox virus	ADZ29155.1 and SNB53780.1	99 and 92
	Monkeypox virus	AAY97225.1	98
	Variola major virus	AAA60767.1	97
	Taterapox virus	ABD97599.1	97
	Camelpox virus	AAL73736.1	96
	Raccoonpox virus	AKJ93661.1 and AKJ93642.1	75 and 45
	Skunkpox virus	AOP31509.1	74
	Volepox virus	AOP31720.1	72
Unclassified *Poxviridae*	Cotia virus	AFB76918.1	70
	Eptesipox virus	ASK51372.1	34
*Centapoxvirus*	Yokapox virus	AEN03759.1	60
	Murmansk poxvirus	AST09387.1	58
*Leporipoxvirus*	Rabbit fibroma virus	AAF18030.1	50
	Myxoma virus	AAF15042.1	50
*Yatapoxvirus*	Tanapox virus	ABQ43480.1	32
	Yaba-like disease virus	CAC21247.1	29
Unclassified *Cervidpoxvirus*	Deerpox virus (W-1170-84)	ABI99004.1	28
Unclassified *Chordopoxvirinae*	Deerpox virus (white-tailed deer poxvirus)	AUI80579.1	28

a Percentage corresponds to the fraction of identical residues between a given protein sequence and the sequence of the M2 protein from vaccinia virus.

### M2-deleted vaccinia virus *in vivo* characterization.

**FIG 10:**
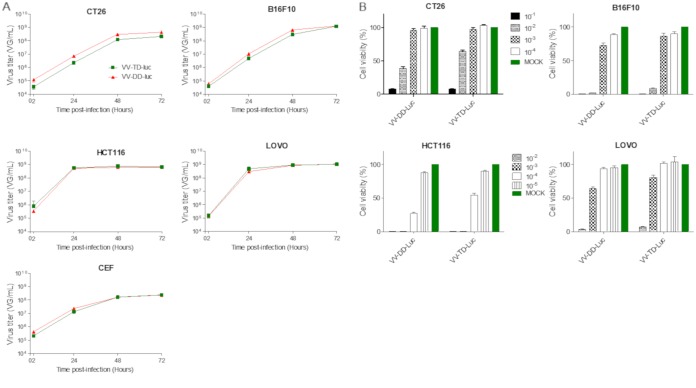
M2L deletion does not impact the replication and the oncolytic activities of the vaccinia virus *in vitro*. (A) Replication of the triple-deleted vaccinia virus (VV-TD-Luc, in green) on CEFs was compared head-to-head with that of the parental double-deleted virus (VV-DD-Luc, in red). Virus replication on CEFs was monitored by qPCR at 2, 24, 48, and 72 h after infection at an MOI of 10^−2^. (B) Oncolytic activity was determined at different MOIs and at 4 (human tumor cells) or 5 (murine tumor cells) days postinfection. The viability of the cells was then measured by trypan blue exclusion and with a cell counter (Vi-Cell; Beckman Coulter). The uninfected cells were used to determine the value for 100% viability. Each measure was performed in triplicate.

**FIG 11:**
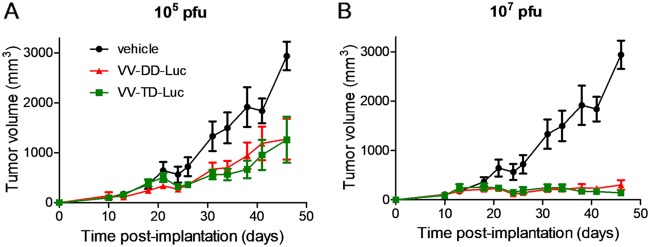
M2L deletion does not affect the oncolytic activities of the vaccinia virus *in vivo*. A total of 5 × 10^6^ HCT116 human tumoral cells was implanted subcutaneously in nude mice. When the tumor size reached 100 to 200 mm^3^ (∼10 days after cell implantation), mice were treated with one intravenous administration of 10^5^ or 10^7^ PFU of doubly (TK^−^ RR^−^) or triply (TK^−^ RR^−^M2L^−^) deleted vaccinia virus encoding firefly luciferase (VV-DD-Luc [red curves] or VV-TD-Luc [green curves], respectively). Tumor volume was measured twice weekly. Each represented value is the mean of the tumor volume (± standard deviation) of 9 animals.

**FIG 12:**
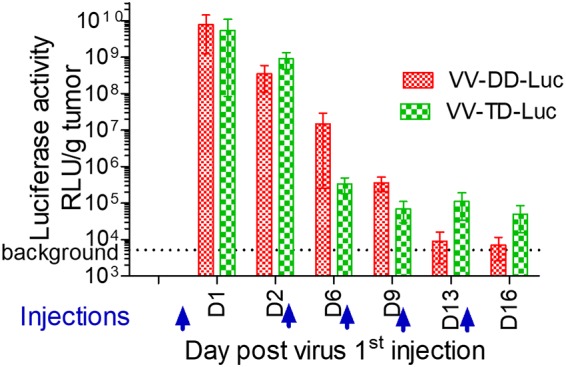
M2L deletion does not affect the transgene expression level *in vivo*. A total of 3 × 10^5^ B16F10 murine tumoral cells was implanted subcutaneously in C57BL/6 mice. When tumor volume reached 20 to 100 mm^3^, 10^7^ PFU of doubly (TK^−^ RR^−^) or triply (TK^−^ RR^−^M2L^−^) deleted vaccinia virus encoding firefly luciferase (VV-DD-Luc or VV-TD-Luc, respectively) was injected intratumorally (day 0) and then at days 3, 6, 9, and 13. At days 1, 2, 6, 9, 13, and 16, three mice/group were euthanized; their tumors were weighed and homogenized, and the luciferase activity was measured according to the recommendations of the provider (Promega) of the kit. The luciferase activity was reported for each time point as a mean of the three measurements. The dashed line represents the background of the luminescence determined from noninjected tumors. RLU, relative light units.

**FIG 13:**
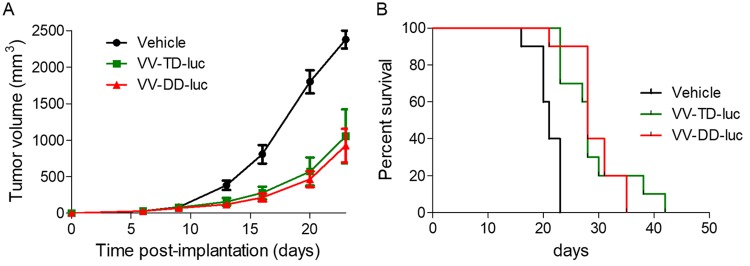
M2L deletion does not affect the antitumoral activity of the vaccinia virus *in vivo*. A total of 3 × 10^5^ B16F10 murine tumoral cells was implanted subcutaneously in C57BL/6 mice. When tumor volume reached 20 to 100 mm^3^, 10^7^ PFU of either VV-DD-Luc (red curves) or VV-TD-Luc (green curves) vaccinia virus was injected intratumorally (day 0) and then at days 3, 6, 9, and 13. The tumor volume was measured twice a week, and the mice were euthanized when this volume reached 2,000 mm^3^. The mean (± error) of the tumor volume (A) or survival (B) are represented versus time.

Almost all of the M2L-deleted recombinant viruses generated were for vaccination purpose, and to our knowledge, none of them were characterized for their oncolytic abilities (20). Therefore, the TD vaccinia virus was compared side by side with its parental DD virus for its replication and oncolytic properties *in vitro* and *in vivo*. The M2L deletion did not have any impact on virus replication in CEFs or in the different tumoral cell lines tested (Fig. 10A). The TD vaccinia virus did not demonstrate any difference of *in vitro* oncolytic activity compared to that of its DD virus counterpart on the three murine and two human tumoral cell lines tested (Fig. 10B). The oncolytic activities of the DD and TD viruses were also compared *in vivo* in a human colorectal xenograft tumor model. In this model, the antitumoral activities of the TD and DD viruses were similar (Fig. 11) (interaction *P* values of 0.189 and 0.082 for doses of 10^5^ and 10^7^ PFU, respectively). Since M2L is probably involved in immunosuppression activities of the vaccinia virus, it was hypothesized that the TD virus could be attenuated and cleared faster than the DD vaccinia virus in an immunocompetent animal. To test this hypothesis, the TD and DD vaccinia viruses expressing luciferase were injected side by side intratumorally (i.t.) in mice bearing a B16F10 tumor implanted subcutaneously. The tumors were harvested at different time points, and luciferase activity was monitored. Figure 12 shows that the luciferase activities over time are quite similar for the two viruses (all *P* values of all time points are >0.3), indicating that the M2L deletion has no impact on vaccinia virus infection and replication, at least in this murine model. Antitumoral activity of the TD versus the DD vaccinia virus was also investigated in the same (B16F10) murine tumor model. The two viruses did not show significant differences in terms of antitumoral activities (*P* value = 0.803) or survival rates (*P* value = 0.998) (Fig. 13A and B). All together these results demonstrated that M2 does not have a major impact on replication or oncolytic activity of the virus either *in vivo* or *in vitro*. Surprisingly, the removal of B7 pathway inhibition in the TD virus did not improve the antitumoral activity of the virus, at least in the rather immuno-resistant B16F10 tumor model.

## DISCUSSION

In this article we show that the vaccinia virus M2 protein is secreted by infected cells as a homo-oligomer that interacts with CD80 and CD86 (also known as B7-1 and B7-2, or B7-1/2) of at least human and murine origins. The M2 binding to CD80 and CD86 inhibits their interaction with both soluble CD28 and CTLA4, their cognate ligands. Unexpectedly, the interaction between soluble PD-L1 and CD80 was not inhibited but was clearly favored by the presence of M2 protein. All activities on the B7-1/2 proteins observed with culture supernatants of infected cells were reproduced with the M2 recombinant purified protein, indicating that, besides M2, there is no other viral or cellular factor involved in interference with the B7-CD28/CTLA4/PD-L1 interactions. The M2L gene is conserved in all orthopoxviruses, and M2L orthologues are found in several other poxvirus families (21). Indeed, the B7 blocking activity was identified in several supernatants of orthopoxvirus-infected cells and was demonstrated also for a recombinant myxoma virus M2 ortholog (i.e., Gp120LP) (22). Although M2 competes with CTLA4/CD28 for the binding to B7-1/2 proteins, it lacks the MYPPPY B7 binding motif found on both molecules. The absence of the B7 binding motif and the fact that, unlike CTLA4/CD28, M2 favors the PD-L1–CD80 interaction indicate that the M2-B7 interface is unique. We hypothesize that the oligomeric M2 proteins might bind several CD80 molecules simultaneously and form a complex that binds PD-L1 with more avidity. The M2 protein is also known for its NF-κB inhibition activity (23, 24) but does not have any known extracellular target and is not even reported as a secreted protein, despite the presence of an identified signal peptide. Moreover, M2 protein was also reported recently to be involved in viral DNA replication that occurs in the cytoplasm of infected cells (25). More studies are needed to decipher how the M2 protein is distributed between extra- and intracellular compartments and which parts of the molecule are involved in each of its very different specific activities (i.e., B7 binding, NF-κB inhibition, and viral DNA replication). Through the activity of M2, CD80 and CD86 join the long list of immune mediators that are known to be modulated or inhibited by vaccinia virus and other poxviruses (13). CD80/CD86 are well-known costimulatory molecules involved in lymphocyte priming, proliferation, and differentiation (26). Indeed, the importance of CD80/CD86 in the immune response against the vaccinia virus in mice has been reported by Salek-Ardakani et al. (27). These molecules generate “signal 2” that drives, with signals 1 (antigen recognition) and 3 (cytokine stimulation), the lymphocytes from a resting to an activated state. By blocking signal 2, vaccinia virus may skew the lymphocyte T fate toward anergy rather activation. This tolerogenic signaling may be reinforced by the fact that M2 might favor the CD80–PD-L1 interaction that has been described to be important for regulatory T cell (Treg) survival and proliferation and for tolerance (28, 29). Together, these immunosuppressive mechanisms, if demonstrated *in vivo*, could contribute to a better tolerance and therefore persistence of the vaccinia virus in the infected host.

Moreover, a Copenhagen vaccinia virus deleted of its M2L gene was generated with the prospect of generating an oncolytic virus with a better immunotherapeutic profile, i.e., improved ability to prime, boost, or sustain an innate and/or adaptive immune response against tumor cells. Indeed, oncolytic vaccinia viruses are considered more and more as immunotherapeutic agents able to fuel the antitumoral response by acting on several key points of the immune response, such as neoantigen release in an immunogenic cell death context (30), dendritic cell maturation (30), and lymphocyte infiltration and activation into tumor (5). These immune effects can even be reinforced by vectorizing a transgene that would positively modulate the immune environment of the infected tumor (8, 9) or, more simply, by combining the oncolytic virus with those immunomodulators. The results presented here do not document the impact of M2L deletion on the profile of the immunological microenvironment of the treated tumor but, rather, focus on the effects of this deletion on viral basics such as replication, transgene expression, and oncolytic activities (both *in vitro* and *in vivo*). The effects of M2L deletion on the immunological properties of the vaccinia virus and on its potential antitumoral benefits will be the subjects of coming studies.

The replication capabilities and several oncolytic properties of the M2L-deleted vaccinia virus and of its parental virus were compared. As expected, the deletion of M2L abolished completely the B7-interfering activities of culture supernatants of infected cells in ELISAs. But other than this activity on B7 pathways, no difference in levels of replication, oncolytic/antitumoral activity, or transgene expression were observed for the M2L-deleted virus versus those of the parental virus both *in vitro* and *in vivo*. These first results indicate that M2L is dispensable not only for the multiplication of the virus *in vitro* as already reported (20, 31) but also *in vivo*, at least in the tumor models tested. The latter result must be tempered by the fact that, as with other oncolytic viruses, the vaccinia virus poorly replicates in murine tumor compared to replication in human tumor (compare the replicative curves of the vaccinia viruses in tumoral murine and human cells lines in Fig. 10A). The B16F10 model is also known to be relatively resistant to many immunomodulators that have proven their antitumoral efficacy in other models. Moreover, the binding on B7 molecules demonstrated that M2 has a weaker affinity for murine CD86 than for its human CD80/CD86 or murine CD80 counterpart. In other words, this mouse model is probably not optimal to investigate the role of the M2L gene in virus persistence, immunogenicity, and antitumoral activity. To really evaluate the benefit of the M2L deletion, experiments on other murine models or other immunocompetent animal models where the vaccinia replication mimics the one observed in human should be conducted.

Taken together, the work presented here paves the way to two new directions of research. The first one will focus on the characterization of the M2 protein as a potential immunosuppressive molecule by documenting its effects on immune cells and on the immune response in whole animals. In addition to the scientific questions that will be answered and raised during this work, one potential output would be the characterization of a potential new immunosuppressive drug. Two drugs, abatacept and belatacept (32, 33), targeting both CD80 and CD86 are currently on the market. These two immunosuppressive drugs are both CTLA4-Fc fusion proteins used in treatment of rheumatoid arthritis (abatacept) or kidney transplantation (belatacept). Abatacept corresponds to the natural human CTLA4 ectodomain fused to an IgG1 Fc, whereas belatacept is a mutated version of CTLA4 fused to the same Fc. Compared to abatacept, belatacept binds CD80 and CD86 with 2- and 4-fold increased affinities, respectively. Our very first results demonstrated that M2 has a better capacity than CTLA4-Fc for blocking the interaction of soluble CD28–B7-1/2. Moreover, and unlike CTLA4-Fc (34), M2 might also able to increase the tolerogenic PD-L1–CD80 interaction. Therefore, M2 has the potential to be a new immunosuppressive drug with improved activity compared to that of the currently marketed molecules.

The second field of investigation will explore the behavior and properties of the M2L-deleted vaccinia virus as a new oncolytic platform able to trigger a more robust antitumoral immune response. This new deletion could reinforce the virus immunostimulatory profile and could synergize with activities of some transgenes acting in the tumor microenvironment on either innate or adaptive pathways of the immune response (i.e., immune checkpoint modulators, cytokines, agonist ligands, etc.). This new virus platform should be studied in animal models where the M2 protein functions are fully operative to assess the impact of M2L deletion on virus replication/persistence and on its antitumoral activity.

## MATERIALS AND METHODS

### Cells and conditions of culture.

The chicken embryo fibroblast cell line DF-1 (ATCC CRL-12203), human colon cancer cell lines LoVo (CCL-229) and HCT 116 (CCL-247), human cervix cancer HeLa (CCL-2) cells, murine melanoma B16F10 (CRL-6475) cells, and murine colon CT26.WT (CRL-2638) cells were obtained from the American Type Culture Collection (ATCC, Rockville, MD, USA). All cell lines were grown in the recommended medium supplemented with 10% fetal bovine serum (FBS). In the case of DF1 and HeLa cells, cells were then washed with phosphate-buffered saline (PBS) before infection and then plated and grown in same medium without FBS. Primary chicken embryo fibroblasts (CEFs) were prepared from chicken embryos obtained from fertilized eggs (Charles River SPAFAS) previously incubated 11 or 12 days at 37°C in a humid atmosphere. Chicken embryos were dissected and treated with a 2.5% (wt/vol) solution of trypsin. CEFs were maintained and infected in virus production serum-free medium (VP-SFM; ThermoFisher) without FBS.

### Proteins and viruses.

FLAG-tagged vaccinia virus M2 protein and myxoma virus Gp120LP protein were produced by transient transfection of HEK293 cells with pVax plasmid (Invitrogen), encoding the recombinant protein, and complexed in Lipofectamine 2000 (Invitrogen). The supernatants of the transfected cells were collected 2 days after transfection, centrifuged, filtered on 0.2-μm-pore-size filter, and stored at −80°C until use. GeneArt (ThermoFisher) produced a FLAG- and His-tagged M2 protein by transient transfection of Expi 293 cells according to its gene-to-protein scale service. The recombinant M2 protein was purified at GeneArt by immobilized metal affinity chromatography from 1 liter of supernatant of transfected cells cultured in suspension for 6 days. The purified M2 protein was aliquoted and stored at −80°C until use.

All other mentioned recombinant proteins (i.e., human and murine costimulation molecules) used were ordered from R&D Systems as Fc fusions with or without a His tag at their C termini. Human CD80-Fc and CD86-Fc were biotinylated in-house using biotinamidohexanoyl-6-aminohexanoic acid N-hydroxysuccinimide ester (Sigma). The anti-hB7-1 (MAb140) and anti-hB7-2 (MAb141) monoclonal blocking antibodies were also ordered from R&D Systems.

All poxviruses used were wild type and have been described in detail in Ricordel et al. (17, 35, 36), except for the vaccinia viruses of Copenhagen strain that were either wild type, doubly deleted of thymidine kinase and ribonucleotide reductase (TK^−^ RR^−^), or triply deleted of TK, RR, and M2L (TK^−^ RR^−^ M2L^−^). Modified vaccinia virus Ankara (MVA) was also used in some experiments. All of these vaccinia viruses were generated at Transgene. The M2L gene deletion, which encompasses 64 nucleotides (nt) upstream of the open reading frame (ORF) and the first 169 codons of the M2L ORF, was performed also at Transgene for this study on DD Copenhagen vaccinia virus by homologous recombination using a transfer plasmid. This transfer plasmid, from pUC18 backbone, contained a left arm (nt 26980 to 27479 of the vaccinia virus genome; GenBank accession number M35027) and a right arm (nt 28051 to 28550) separated by an expression cassette encoding the fusion of the selection markers enhanced green fluorescent protein/xanthine-guanine phosphoribosyl transferase (EGFP/GPT) under control of the vaccinia pH5R promoter. Upstream of the right arm, a repeat of the left arm was introduced to favor the further interarm recombination and therefore the elimination of the selection markers.

This plasmid was transfected by electroporation into CEFs infected by vaccinia virus encoding luciferase (RR^−^ TK^−^ Luc) using an Amaxa Nucleofector. A recombinant virus was isolated by EGFP/GPT selection. The deletion of M2L and insertion of the EGFP/GPT cassette were confirmed by PCR analysis. The EGFP/GPT selection cassette was removed by two passages of the recombinant virus on CEFs without selection. The deletion of the M2L gene was verified by PCR and sequencing. The M2L-deleted virus has the genotype TK^−^ RR^−^ M2L^−^ Luc (VV-TD-Luc) and its parental virus has the genotype TK^−^ RR^−^ Luc (VV-DD-Luc).

All vaccinia viruses were produced on CEFs. Three days after infection, the crude harvest containing infected cells and culture supernatant was recovered and stored at −20°C until use. Prior to purification and in order to release viral particles, this suspension was homogenized using a homogenizing mixer equipped with an in-line chamber. Large cellular debris was then eliminated by depth filtration using filters of 5-μm pore size. The clarified viral suspension was subsequently concentrated and diafiltered with formulation buffer (50 g/liter saccharose, 50 mM NaCl, 10 mM Tris, 10 mM glutamate-Na, pH 8.0) by using tangential flow filtration and 0.2-μm-pore-size hollow-fiber microfiltration filters. Finally, the purified virus was further concentrated using the same tangential flow filtration system, aliquoted, and stored at −80°C until use.

### *In vitro* replication and oncolytic activities.

For replication assays, CT26 or B16F10 cells at 2 ×10^5^ cells per well and HCT116 or LoVo cells at 8 × 10^5^ cells per well were infected at an MOI of 10^−2^ in suspension and then plated in six-well culture dishes with 2 ml of culture medium. For CEFs, 1 × 10^6^ cells per well were plated and infected at an MOI of 10^−2^. At the time points indicated in the legend to Fig. 10, cells and medium were harvested and frozen at −80°C until use. Cell suspensions were thawed and sonicated, and 100 μl was treated with 5 units of Benzonase (Novagen) to eliminate nonencapsidated DNA. Benzonase was then inactivated by addition of EDTA (27 mM final concentration), and the virus capsids were disrupted by addition of an equal volume of 20 mM Tris, 10 mM EDTA, and 1% SDS, pH 7.4, followed by 160 μg of proteinase K (Qiagen) and an incubation of 30 min at 65°C. Proteinase K was then inactivated by an incubation of 15 min at 95°C. The samples were stored at −20°C until quantitative PCR (qPCR) analysis. The qPCR assay was a relative quantification using purified and quantified MVA DNA as a standard. A Quantitect Multiplex PCR kit from Qiagen was used to perform the qPCRs on a 7500 real-time system from Applied Biosystem (SDS software, version 2.0.6.) as described previously (9).

For viability assays, CT26 or B16F10 cells at 2 × 10^5^ cells per well or HCT116 or LoVo cells at 8 × 10^5^ cells per well were infected at an MOI of 10^−1^ to 10^−4^ (murine cells) or 10^−2^ to 10^−5^ (human cells) in suspension and then plated in six-well culture dishes with 2 ml of culture medium. Each condition was performed in triplicate. At 4 days (HCT116 and LoVo) or 5 days (CT26 and B16F10) after infection, cell viability was determined by trypan blue exclusion with a cell counter (Vi-Cell; Beckman Coulter). The condition of untreated cells was used to set the value for 100% viability.

### ELISA for B7 binding.

Ninety-six-well plates (Nunc-Immuno plates; Medisorp) were coated overnight at 4°C with 100 μl (at 0.5 or 1 μg/ml) of either of the B7 proteins, CTLA4, or CD28 in coating buffer (50 mM Na-carbonate, pH 9.6). Microplates were washed by PBS–0.05% Tween 20 and saturated by 200 μl of blocking solution (PBS, 0.05% Tween 20, 5% nonfat dry milk [Bio-Rad]). All dilutions were made in blocking solution. Samples (100 μl) were added to each well in triplicate and in 2-fold serial dilutions for some experiments (binding curves). Microplates were then incubated with 100 μl of a horseradish peroxidase (HRP)-conjugated anti-Flag antibody (Sigma) diluted 10,000-fold. Microplates were then incubated with 100 μl/well of 3,3′,5,5′-tetramethylbenzidine (TMB; Sigma), and the reaction was stopped with 100 μl of 2 M H_2_SO_4_. Absorbance was measured at 450 nm with a plate reader (Infinite M200 PRO; Tecan). The absorbance values were transferred into the software program GraphPad Prism for analysis and graphic representation.

### Competition ELISA.

For competition ELISAs, experimental conditions and solutions, not otherwise specified, were identical to the ones described above. Refer to each figure legend for the cells used and infection conditions (MOI and time postinfection) that generated the different supernatants tested. For CTLA4-CD80/CD86, CD28-CD80/CD86, and PD-L1–CD80 competition assays, 100 μl of CTLA4-Fc, CD28-Fc, and PD-L1–Fc was coated at 0.25 μg/ml (CTLA4), 2 μg/ml (CD28), or 1 μg/ml (PD-L1). Samples were added and diluted (2-fold serial dilution) in blocking solution containing a constant concentration of either CD80-Fc (biotinylated or not; from 10 to 250 ng/ml) or CD86-Fc (biotinylated or not; from 100 to 500 ng/ml), depending of the interaction. Either anti-His-HRP (Qiagen) at 1/5,000 or streptavidin-HRP (Southern Biotech) at 1/1,000 was used as a conjugated reagent. The plates were further treated, and results were analyzed as described above.

### Western blotting.

Samples from supernatants of transfected or infected cells were treated either directly or after a 20-fold concentration using a Vivaspin 20 30,000-molecular-weight-cutoff (MWCO) concentrator (Sartorius). Twenty-five microliters of sample was prepared in Laemmli buffer with (reducing condition) or without (nonreducing condition) 5% beta-mercaptoethanol. After electrophoresis on Criterion TGX 4 to 15% stain-free gels (Bio-Rad), the proteins were transferred to polyvinylidene difluoride (PVDF) membrane (Transblot Turbo System). An IBind Flex Western system (Invitrogen) was used for the protein/antibody incubations and washes. Blots were probed either with 2.5 μg/ml of CD80-Fc, CD86-Fc, or CTLA4-Fc or with anti-Flag-HRP at a 1/1,000 dilution. For CD80-Fc, CD86-Fc, and CTLA4-Fc, an anti-human Fc-HRP antibody (Bethyl) at 1/3,000 was used as the conjugated antibody. The 1× iBind Flex Solution was used to block, dilute the antibodies, and wash and wet the iBind Flex Card. Immune complexes were detected using Amersham ECL Prime Western blotting reagents. Chemiluminescence was recorded with a ChemiDOC XRS molecular imager (Bio-Rad).

### Affinity chromatography.

Supernatants of CEFs infected (MOI of 0.05) by either MVA or vaccinia virus Copenhagen (TK^−^ RR^−^) were collected at 72 h postinfection. The supernatants supplemented with 0.05% Tween 20 were concentrated ∼20-fold using a Vivaspin 20 30,000-MWCO concentrator (Sartorius). Streptavidin-covered magnetic beads (GE Health Care) were coated with either an irrelevant monoclonal biotinylated antibody (chCXIIG6-Biot), CTLA4-Fc-Biot, or CD86-Fc-Biot. Four milliliters of concentrated supernatants (MVA and vaccinia virus Copenhagen) was incubated with 24 μl of chCXIIG6-streptavidin beads to remove most of the nonspecific binding. The flowthrough of this first incubation was split into two equal parts and incubated with either CTLA4-Fc-Biot-streptavidin beads or CD86-Fc-Biot-streptavidin beads to yield the four following arms: MVA supernatant plus CTLA4 beads (MVA/CTLA4), MVA supernatant plus CD86 beads (MVA/CD86), vaccinia virus plus CTLA4 bead supernatant (VV/CTLA4), and vaccinia virus plus CD86 beads (VV/CD86). The beads were extensively washed with PBS and 0.05% Tween 20, followed by PBS, and bound proteins were eluted by two successive additions of 50 μl of 0.1 M acetic acid neutralized immediately by addition of 4 μl of 2 M Tris base. The two elutions were then pooled for MS analysis.

### Mass spectrometry analysis.

Ten or 20 μl of sample was evaporated and submitted to reduction by solubilization in 10 μl of 10 mM dithiothreitol (DTT) in 25 mM NH_4_HCO_3_ (1 h at 57°C). Reduced cysteine residues were alkylated in 10 μl of 55 mM iodoacetamide in 25 mM NH_4_HCO_3_ for 30 min at room temperature in the dark. The trypsin (12.5 ng/μl) (V5111; Promega), freshly diluted in 25 mM NH_4_HCO_3_, was added to the sample in a 1:100 (enzyme/protein) ratio to a final volume of 30 μl and incubated for 5 h at 37°C. The activity of the trypsin was inhibited by acidification with 5 μl of H_2_O–5% trifluoroacetic acid (TFA). Samples were analyzed on a nano-ultraperformance liquid chromatography (nano-UPLC) system (nanoAcquity; Waters) coupled to a quadrupole-Orbitrap hybrid mass spectrometer (Q-Exactive Plus; ThermoFisher Scientific, San Jose, CA). The UPLC system was equipped with a Symmetry C_18_ precolumn (20 by 0.18 mm, 5-μm particle size; Waters, Milford, MA, USA) and an Acquity UPLC BEH130 C_18_ separation column (75 μm by 200 mm, 1.7-μm particle size; Waters). The solvent system consisted of 0.1% formic acid in water (solvent A) and 0.1% formic acid in acetonitrile ([ACN] solvent B). Two microliters of each sample was injected. Peptides were trapped during 3 min at 5 μl/min with 99% A and 1% B. Elution was performed at 60°C at a flow rate of 400 nl/min, using a 79-min linear gradient from 1% to 35% B. To minimize carryover, a column wash (50% ACN during 20 min) was included in between each sample in addition to a solvent blank injection, which was performed after each sample.

The Q-Exactive Plus MS was operated in positive-ion mode with source temperature set to 250°C and spray voltage set to 1.8 kV. Full-scan MS spectra (300 to 1,800 *m/z*) were acquired at a resolution of 140,000 at *m/z* 200, a maximum injection time of 50 ms, and an automatic gain control (AGC) target value of 3 × 10^6^ charges with the lock-mass option being enabled (445.12002 *m/z*). Up to 10 most intense precursors per full scan were isolated using a 2-*m/z* window and fragmented using higher-energy collisional dissociation (HCD; normalized collision energy of 27 eV), and dynamic exclusion of already fragmented precursors was set to 60 s. MS/MS spectra were acquired with a resolution of 17,500 at *m/z* 200, a maximum injection time of 100 ms, and an AGC target value of 1 × 10^5^ charges. The system was fully controlled by XCalibur software (version 3.0.63; ThermoFisher Scientific). MS/MS data were searched against a Gallus gallus and vaccinia virus UniProt database-derived combined target-decoy database (containing 33,939 target sequences plus the same number of reversed decoy sequences) using Mascot (version 2.5.1; Matrix Science, London, England). The target proteins hCTLA4, hCD86, and hCXIIG6 (human IgG1) and target-decoy were manually added to the database. The database including common contaminants (human keratins and porcine trypsin) was created using an in-house database generation toolbox. The following parameters were applied: one missed cleavage by trypsin and variable modifications oxidation of methionine [+16 Da], carbamidomethylation of cysteine [+57 Da] were considered. The search window was set to 25 ppm for precursor ions and 0.07 Da for fragment ions. Mascot result files (.dat) were imported into Proline software (http://proline.profiproteomics.fr/), and proteins were validated on pretty rank equal to 1, 1% false discovery rate (FDR) on peptide spectrum matches based on an adjusted E value, at least one specific peptide per protein, 1% FDR on protein sets, and Mascot modified MudPIT (multidimensional protein identification technology) scoring.

### Flow cytometry.

The ability of M2 to interact with cell surface CD80 and CD86 was studied in flow cytometry-based binding and competition assays using CD80- and CD86-positive human Hodgkin lymphoma-derived KM-H2 cells (DSMZ). KM-H2 cells were first incubated with fluorescent reactive dye from a Live/Dead Fixable Violet Dead Cell Stain kit (Invitrogen) at a 1/1,000 dilution and washed with PBS. For CD80 and CD86 staining, cells were then incubated with anti-human CD80-phycoerythrin (CD80^PE^; clone 2D10), anti-human CD86-peridinin chlorophyll protein-Vio700 (CD86^PerCP-Vio700^; clone FM95), or the respective fluorochrome-conjugated isotype-matched control (IS5-21F5) from Miltenyi Biotec at a 1/10 dilution. For assessing the binding of M2, cells were incubated with 0.1, 1, or 10 μg/ml purified recombinant FLAG-tagged M2 or 10 μg/ml irrelevant FLAG-tagged protein as a negative control and washed with PBS, and M2 was detected with 1 μg/ml anti-FLAG tag-PE (L5) from BioLegend. For assessing the blocking activity of cell culture supernatants, KM-H2 cells were coincubated with 100 ng/ml human CTLA4-Fc (R&D Systems) and supernatant at a 1/1.25 dilution. After cells were washed, the binding of CTLA4-Fc to KM-H2 cells was detected using 5 μg/ml anti-human IgG Fc^PE^ (HP6017) from BioLegend. All incubation steps were carried out on ice for 30 to 45 min. Antibodies, proteins, and the violet fluorescent reactive dye were diluted in PBS. Fluorescence intensity was measured on a Navios flow cytometer (Beckman Coulter). Data were analyzed using Kaluza, version 1.3, software (Beckman Coulter).

### *In vivo* experiments.

All animal protocols were carried out according to standard operating procedures of the Federation of European Laboratory Animal Science Associations and were been approved by the French Research and Education Ministry (APAFIS number 7049-2016110401034662).

Female 6-week-old C57BL/6 or nude mice were kept in accordance with policies on animal research at the Faculté de Pharmacie of Illkirch-Graffenstaden. *In vivo* projects were approved by the regulatory authorities for animal welfare.

### (i) Syngeneic model (B16F10) for antitumoral activity and transgene expression assessments.

C57BL/6 mice were inoculated subcutaneously (s.c.) with 3 × 10^5^ B16F10 melanoma cells into the right flank. After 7 days, tumors were measured, and mice were randomly distributed into treatment groups. Virus (10^7^ PFU/50 μl of either VV-TD-Luc or VV-DD-Luc) was injected intratumorally at days 0, 3, 6, 9, and 13. Tumor dimensions were measured twice a week with calipers, and their volumes were calculated using the formula (π/6)(length × width^2^). The animals were euthanized when their tumor volumes reached 2,000 mm^3^. For luciferase activity determination, 3 or 4 mice per group were sacrificed at days 1, 2, 6, 9, 13, and 16. Tumors were harvested and weighed. Cells were lysed with passive lysis buffer (E1941; Promega) and OctoMACS technology (Miltenyi). Supernatants were harvested and frozen at −80°C until luciferase activity determination. Luciferase activity was determined according to the manufacturer’s protocol (E2920; Promega). Luciferase specific activity was reported as the enzyme activity divided by the mass of the tumor.

### (ii) Xenograph model (human colorectal HCT116 tumoral cells).

Nude mice were inoculated s.c. with 5 × 10^6^ HCT116 human colorectal tumoral cells. After 10 days, when tumor volume reached 100 to 200 mm^3^, mice were randomly distributed into treatment groups. Mice were then treated once intravenously (via tail vein) with either the vehicle or VV-TD-Luc or VV-DD-Luc at a dose of 10^5^ or 10^7^ PFU (9 mice per group). Tumor size was measured twice weekly using calipers. Tumor volumes were calculated in cubic millimeters using the formula (π/6)(length × width^2^). The animals were euthanized when their tumor volumes reached 3,000 mm^3^.

### Statistical methods.

Analyses were conducted using SAS, version 9.4. Tests were performed at the level of 5%. 

### (i) Tumor size evolution.

Tumor diameter (expressed in millimeters) was derived from tumor volume calculated with length and width (expressed in cubic millimeters) using the following formula: diameter = 2 × [(3 × volume)/(4π)]^1/3^.

A repeated mixed model was built using tumor diameter as response. Treatment group, day (as continuous), and the interaction between both factors were considered fixed effects. Mouse was considered a random effect, and a repeated measure over time was considered with a spatial power structure for variance-covariance. If some effects were found significant, *post hoc* comparisons were done with Tukey’s multiplicity adjustment. When comparisons were performed with vehicle, a Dunnett’s correction was applied.

### (ii) Survival analysis.

Groups were compared with a log rank test, and, if significant, *post hoc* pairwise comparisons were done with Tukey’s multiplicity adjustment.

### (iii) *In vivo* luciferase level comparisons.

A general linear model was used to evaluate the differences between the two groups (VV-DD-Luc versus VV-TD-Luc), with group and time point as factors and interaction of both.
